# Efficacy and Safety of Sufentanil Infusion for Postoperative Analgesia in Cancer Surgery: A Retrospective Cohort Study

**DOI:** 10.7759/cureus.38993

**Published:** 2023-05-14

**Authors:** Sofia Dias, Sofia Trovisco, Inês Neves, Lina Miranda, Rui Valente

**Affiliations:** 1 Department of Anesthesiology and Intensive Medicine, Instituto Português de Oncologia do Porto Francisco Gentil, EPE, Porto, PRT

**Keywords:** multimodal analgesia, surgery, cancer, parenteral infusions, sufentanil

## Abstract

Background

Opioids have long been the cornerstone of drugs used for perioperative analgesia. Sufentanil has an advantageous pharmacological profile for its use in continuous intravenous (IV) infusion, yet remains poorly described. Our institution has implemented analgesia protocols with IV sufentanil infusions for cancer surgery with appropriate monitoring. The aim of this study was to evaluate the efficacy and safety of IV sufentanil infusion.

Methods

A single-center retrospective cohort study was conducted through the analysis of patients’ records and the acute pain service database. Inclusion criteria were adult patients admitted for elective cancer surgery and with postoperative IV sufentanil infusion during one year period. Descriptive and inferential statistical analysis was performed by using Software SPSS Statistics (IBM Corp., Armonk USA): Kruskal-Wallis, Mann-Whitney, Chi-square and Fisher tests; Bonferroni chi-square residual analysis, binary logistic regression; p<0.05.

Results

The study population of 304 patients had a median age of 66 years (22-91) and 229 (75.3%) were men. 38 (12.5%) were chronic opioid users. Head and neck/otorhinolaryngology (ORL) surgery was performed in 155 (51.0%) and abdominopelvic surgery in 123 (40.5%). The median days of IV sufentanil infusion were 2 (1-13). At rest and with movement, analgesia was considered good, i.e., over 90% of patients with visual analogue scale (VAS) pain score ≤ 3. We found that patients submitted to musculoskeletal surgery had higher VAS pain scores; this group also presented older patients with higher American Society of Anesthesiologists (ASA) physical status classification and more chronic opioid users (p<0.05).

144 patients (47.4%) had at least one adverse effect related to IV sufentanil infusion, notably transient and not requiring any specific treatment. These patients were older and had longer infusion periods (p<0.05). 237 (98.3%) of the adverse effects occurred during the first 3 days and the most common were: sedation (n=104, 42.8%), hypotension (n=32, 13.2%), hypoxemia (n=31, 12.8%) and nausea/vomiting (n=25, 10.3%). The reported incidence of respiratory depression was 2.9% (n=9), with three patients (1%) requiring advanced treatment.

Conclusion

Multimodal analgesic protocols with IV sufentanil infusions provided good postoperative analgesia for head and neck/ORL and abdominopelvic cancer surgeries. The adverse effects associated with the IV sufentanil infusions were mild and mainly managed with opioid dose reductions. Our study showed that this approach can be a safe option for postoperative multimodal analgesia in cancer surgery with appropriate monitoring in high-dependency units.

## Introduction

Opioids have long been the cornerstone of perioperative analgesia, assuming an important role in controlling moderate to severe acute pain [1,2] as a part of a multimodal anesthesia approach. Formally, they act upon opioid receptors, in particular µ, at a central and peripheral level. While they can effectively relieve pain, opioid administration carries some risks. Common side effects of opioid administration include sedation, dizziness, nausea, vomiting, constipation, physical dependence, tolerance, and respiratory depression [3].

Opioid-free anesthesia is growing in interest, firstly motivated by the public health crisis resulting from the opioid epidemic in the USA and by a possible association with cancer recurrence, still lacking proper evidence [4]. Currently, acute postoperative pain remains a problem in more than 80% of patients [5] which brings other significant risks including chronic postoperative pain affecting 10% of patients [6]. Recent evidence-based guidelines recommend against routine basal infusion of opioids in opioid-naïve patients, with moderate-quality evidence, considering a greater risk of side effects and lack of advantage in pain control [7]. Consequently, postoperative opioid infusion use has been poorly described and used for short periods [8,9].

Sufentanil, a synthetic analog of fentanyl, is a highly lipophilic opioid, with a quick onset of action and short context-sensitive half-time, it is 10 times more potent than fentanyl and has a stable hemodynamic profile [3,10]. At the same time, it has a high therapeutic index and is less expensive when compared to remifentanil [3,11]. Its rapid distribution and high clearance suggest clinical advantages of sufentanil infusions, preventing accumulation when given for a long period of time. However, in clinical anesthesia, its use has been essentially described as an adjuvant to local anesthetics in neuraxial anesthesia.

Over more than ten years, our institution has been implementing postoperative multimodal analgesia protocols with IV sufentanil infusions, mainly for patients undergoing head and neck/ORL and abdominal cancer surgery.

The aim of this study was to evaluate retrospectively the efficacy and safety of IV sufentanil infusions for postoperative analgesia in patients who underwent elective surgery over one year in a European cancer center.

## Materials and methods

Study design and participants

A retrospective cohort study was conducted with the approval of the Ethics Committee of our Institution (approval number 162/021) and included all adult patients admitted for elective surgery with postoperative IV sufentanil analgesic protocols, between January to December 2019. Data were collected from the patients’ electronic records (medical and nursing) and the acute pain service database.

In our institution, IV sufentanil infusions, prepared in a 5 mcg/mL dilution, are initiated intraoperatively or at the end of surgery, and titrated according to patient needs, from 2.5 to 20 mcg/h (1 - 4 mL/h). These infusions are prescribed in a multimodal analgesic strategy, combined with paracetamol every 6h and with or without parecoxib every 12h during 72h. After discharge from Post Anaesthesia Care Unit (PACU), these patients maintained their vigilance and monitoring in high-dependency units. All patients with analgesic protocols that included IV sufentanil infusions received a daily visit by an anaesthesiologist of the Acute Pain Service until deescalating analgesia strategy, in order to maximize efficacy and safety.

Study variables

Preoperative data collected included demographic variables, American Society of Anesthesiologists (ASA) physical status classification, previous opioid consumption, comorbidities, and type of surgery. Regarding the postoperative period, we collected sufentanil IV protocol used (with or without parecoxib), the number of days with sufentanil infusion, pain at rest and with movement evaluated by the verbal rating scale (VRS) and converted to visual analogue scale (VAS): none (VAS=0), mild (VAS 1-3), moderate (VAS 4-6) or severe (VAS 7-10) pain. Of note that the correspondence between the various pain rating scales allows us to adapt to the patient’s communication skills. VAS is difficult to apply in the immediate postoperative period and in the elderly. For this reason, our institution adopted a simpler scale (VRS) for pain assessment over 20 years.

Adverse effects were registered during the period with the IV sufentanil infusion until its discontinuation.

Pain at rest and with movement was analysed considering the median of days with IV sufentanil infusion. Additionally, to analyse the efficacy of analgesia, it was categorised as good (with records of pain absence or mild pain; i.e. VAS≤3), reasonable (at least one record of moderate pain; i.e. VAS 4-6) or bad (at least one record of severe pain; i.e. VAS≥7) during median IV sufentanil infusion period.

Adverse effects were registered by day for every patient since the postanaesthetic care unit stay until the end of sufentanil infusion. We categorized adverse effects into four groups: a) respiratory (hypoxemia (peripheral oxygen saturation SpO2 <90% or partial pressure of oxygen paO2 <60mmHg), bradypnea (respiratory frequency (RF)<10 bpm), hypercapnia (partial pressure of carbon dioxide (paCO2)>50mmHg)); b) cardiovascular (bradycardia (heart rate (HR)<60bpm), hypotension (mean arterial pressure (MAP)<60mmHg), cardiorespiratory arrest); c) neurologic (sedation (without spontaneous ocular opening), agitation); d) others (uncontrolled pain; nausea or vomiting; pruritus; urinary retention). Respiratory depression was defined as bradypnea or hypoxemia or hypercapnia and it was measured as the percentage of patients who suffer at least one of these events combined with sedation, during IV sufentanil infusion.

Statistical analysis

For descriptive analysis, categorical variables were expressed as absolute and relative frequencies (%), and continuous variables were expressed as mean ± standard deviation (SD) or median (range) for variables with skewed distributions. Normality was assessed by using skewness and kurtosis measures. Time to adverse effects’ occurrence during the IV sufentanil infusion period was described by the Kaplan-Meir method. For inferential analysis, the association between categorical and continuous variables was explored by the Kruskal-Wallis test, followed by the Mann-Whitney test between pairs of variables. Association between categorical variables was inferred by Chi-square or Fisher tests, followed by chi-square residual analysis to explore significant results. Binary logistic regression was adjusted considering age and days with IV sufentanil infusion regarding adverse effects related to IV sufentanil infusion. Statistical analysis was performed using Software SPSS Statistics (v.26; IBM Corp., Armonk, USA) and statistical significance was set at p <0,05, 2-tailed.

## Results

**Figure 1:**
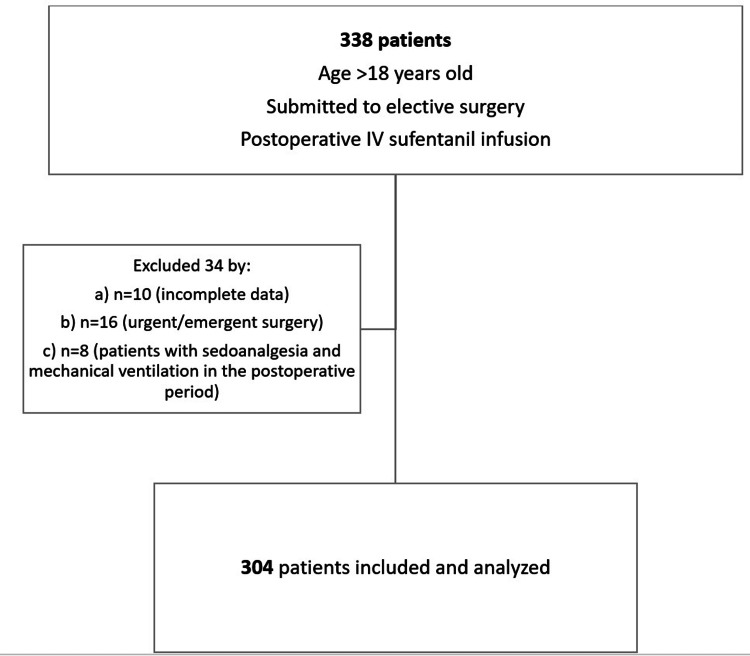
Patient inclusion process

A total of 338 patients verified our inclusion criteria in the study period; of these, 34 were excluded and the remaining 304 were further analyzed (Figure 1).

The demographics and preoperative variables of 304 patients under IV sufentanil infusion are shown in Table 1. 229 (75.3%) were male with a median age of 66 (22-91) years. The majority were classified as ASA III (n= 202, 66,4%). 38 (12.5%) patients had previous opioid consumption. Head and neck/ORL surgery (n=155, 51%) and abdominopelvic surgeries (n=123, 40,5%) were the most prevalent. Regarding IV sufentanil infusion-based protocols, patients were under this infusion for a median of two days (1-13), and in 195 (64.1%) of patients, this protocol was not combined with a prescription of non-steroidal anti-inflammatory drug (NSAID).

**Table 1:** Patients’ demographics, preoperative variables, surgery, and IV sufentanil infusion protocol type and duration. American Society of Anaesthesiology (ASA) physical status classification; Otorhinolaryngology (ORL)

	Total (n=304)	
Age (median (range), years)	66 (22-91)	
Gender (n %)		
Female	75	(24.7%)
Male	229	(75.3%)
ASA physical status (n %)		
II	94	(30.9%)
III	202	(66.4%)
IV	8	(2.6%)
Previous opioid consumption (n %)		
Yes	38	(12.5%)
No	266	(87.5%)
Type of surgery (n %)		
Abdominopelvic	123	(40.5%)
Head and neck/ORL	155	(51.0%)
Thoracic	18	(5.9%)
Musculoskeletal	4	(1.3%)
Spine	4	(1.3%)
IV sufentanil infusion-based protocol (n %)		
With NSAIDs	109	(35.9%)
Without NSAIDs	195	(64.1%)
Days with IV sufentanil infusion (median (range))	2 (1-13)	

The duration of IV sufentanil infusion varies with the type of surgery, although not statistically significant (Table 2). According to this, we analyzed the efficacy of analgesia in relation to the median of days with IV sufentanil infusion.

**Table 2:** Descriptive analysis of patients with IV sufentanil infusion and efficacy of analgesia by type of surgery. The efficacy of analgesia was classified as good (with records of VAS≤3), reasonable (at least one record of VAS 4-6), or bad (at least one record of VAS≥7) during the median IV sufentanil infusion period. American Society of Anaesthesiologists (ASA) physical status classification; Otorhinolaryngology (ORL); Visual Analogue Scale (VAS); Non-steroidal anti-inflammatory drug (NSAID); KW: Kruskal-Wallis; MW: Mann-Whitney. Statistically significant: *p<0,05, comparing to patients submitted to spine surgery; **p<0,005; ^#^p<0,003.

Type of surgery	Abdominopelvic (n=123)		Head and neck/ORL (n=155)		Thoracic (n=18)		Musculoskeletal (n=4)		Spine (n=4)		p-value	Test
Age (median (range), years)	67 (28-86)		63 (33-91)		71 (22-79)		81.5 (69-84)*		45 (44-61)		0.003	KW
Gender (n %)												
Female	42	(34.1%)	28	(18.1%)	4	(22.2%)	1	(25%)#	0		0.025	Fisher
Male	81	(65.9%)	127	(81.9%)	14	(77.8%)	3	(75%)#	4	(100%)		
ASA physical status (n %)												
II	38	(30.9%)	49	(31.8%)	5	(27.8%)	0		2	(50%)	0.005	Fisher
III	79	(64.2%)	106	(67.5%)	13	(72.2%)	2	(50%)	2	(50%)		
IV	6	(4.9%)	0		0	(59.1)	2	(50%)#	0			
Previous opioid consumption (n %)												
Yes	11	(8.9%)	21	(13.5%)	0		4	(100%)**	2	(50%)	0.000	Fisher
No	112	(91.1%)	134	(86.5%)	18	(100%)	0	**	2	(50%)		
IV sufentanil infusion-based protocol (n %)											0.000	Fisher
With NSAIDs	4	(3.3%)**	96	(61.9%)**	7	(38.9%)	0		3	(75%)		
Without NSAIDs	119	(96.7%)**	59	(38.1%)**	11	(61.1%)	4	(100%)	1	(25%)		
Days with IV sufentanil infusion (median (range))	2 (1-13)		2 (1-6)		1 (1-4)		1.5 (1-4)		2 (1-3)		0.106	KW
Efficacy of analgesia												
*At rest* (n %)												
Good	117	(95.1%)	152	(98.1%)	17	(94.4%)	0	#	4	(100%)	0.000	Fisher
Reasonable	5	(4.1%)	3	(1.9%)	1	(5.6%)	3	(75%)#	0			
Bad	1	(0.8%)	0		0		1	(25%)#	0			
*At movement* (n %)												
Good	99	(80.5%)	145	(93.5%)	13	(72.2%)	0	#	4	(100%)	0.000	Fisher
Reasonable	23	(18.7%)	10	(6.5%)	5	(27.8%)	3	(75%)#	0			
Bad	1	(0.8%)	0		0		1	(25%)#	0			

**Figure 2:**
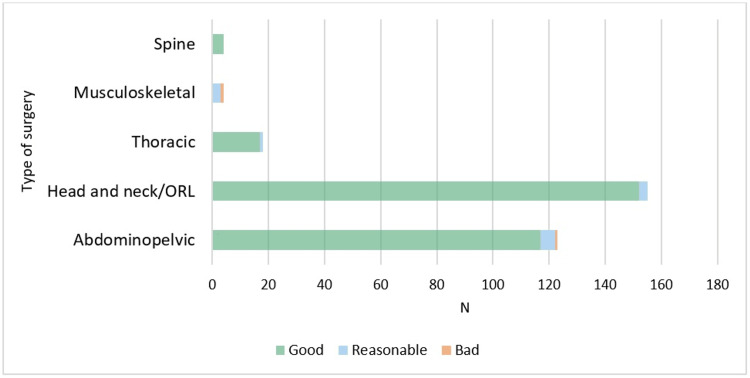
Efficacy of analgesia at rest with IV sufentanil infusion, by type of surgery. The efficacy of analgesia was classified as good (with records of VAS≤3), reasonable (at least one record of VAS 4-6), or bad (at least one record of VAS≥7) during the median IV sufentanil infusion period. Visual Analogue Scale (VAS); Otorhinolaryngology (ORL).

**Figure 3:**
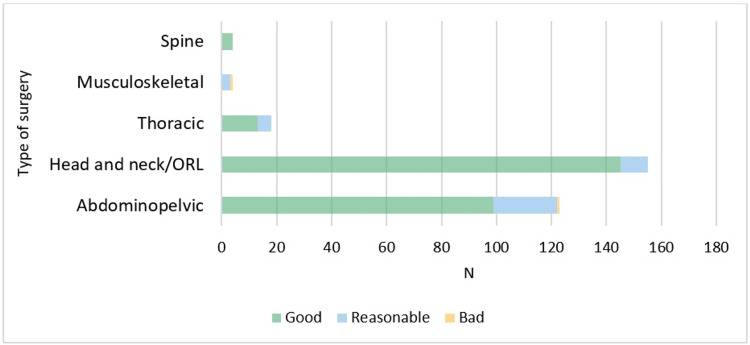
Efficacy of analgesia at movement with IV sufentanil infusion, by type of surgery. The efficacy of analgesia was classified as good (with records of VAS≤3), reasonable (at least one record of VAS 4-6), or bad (at least one record of VAS≥7) during the median IV sufentanil infusion period. Visual Analogue Scale (VAS); Otorhinolaryngology (ORL).

Excluding musculoskeletal and spine surgery, our results show good efficacy of analgesia in the majority of patients (i.e. over 90% of patients with VAS≤3) considering pain at rest and pain with movement with IV sufentanil infusion, clearly seen in Figures 2, 3.

Comparing patients by the type of surgery, in relation to preoperative variables and days of infusion, there were some statistical differences (Table 2). Further analysis showed that these statistically significant differences in preoperative variables were especially in patients submitted to musculoskeletal surgery in which the efficacy of analgesia was worse (p<0.003). These patients were older (p<0.05), presented a higher prevalence of previous opioid consumption (p<0.005), and had a higher ASA physical status classification (p<0.003) in comparison to other groups. Patients submitted to abdominopelvic surgery showed a higher prevalence of IV sufentanil infusion-based protocol without a prescription of NSAID regarding patients submitted to head and neck surgery (p<0.005). 

**Figure 4:**
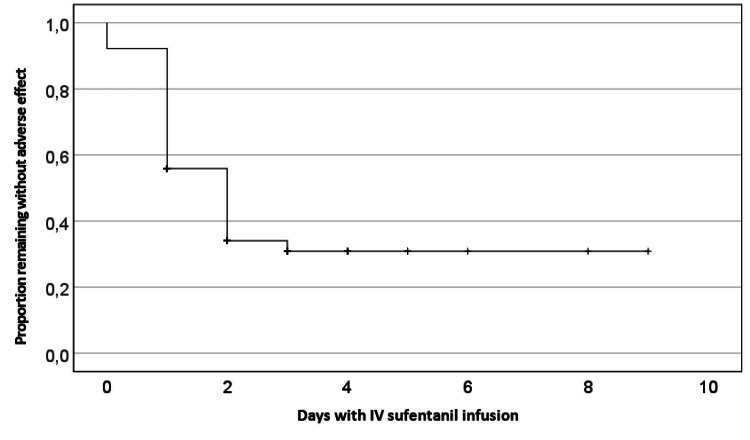
Kaplan-Meier curve for adverse effect-free probability during IV sufentanil infusion period (days). Patients were censored on the last day of infusion.

**Figure 5:**
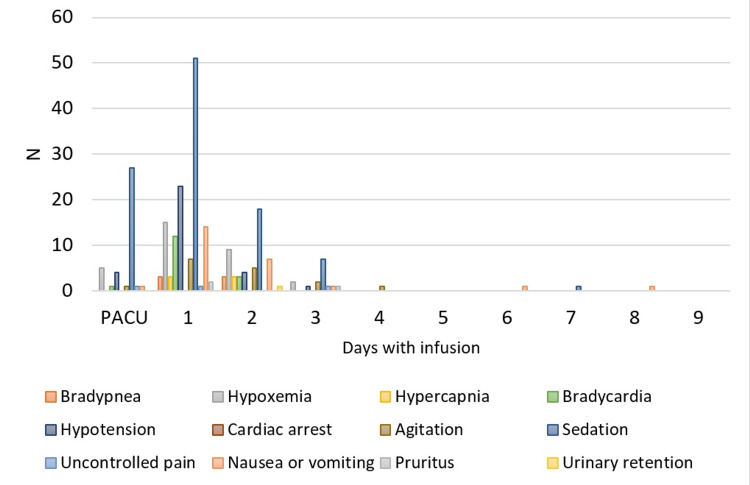
Adverse effects during IV sufentanil infusion period. Post-Anaesthesia Care Unit (PACU)

144 patients (47.4%) had at least one adverse effect during IV sufentanil infusion (Figure 4), given a total of 243 adverse effects described in Figure 5, per day, that sometimes occurred simultaneously. Comparing patients with to the ones without adverse effects (Table 3), these are older (adjusted p=0.003; OR 1.031 with 95% CI (1.011-1.052)) and present longer periods (adjusted p=0.021, OR 1.224; 95% CI (1.031-1.453)) under IV sufentanil infusion. There was no difference regarding gender, ASA physical status classification, type of surgery, previous opioid consumption, and efficacy of analgesia.

**Table 3:** Comparison between patients with and without adverse effects related to IV sufentanil infusion. The efficacy of analgesia was classified as good (with records of VAS≤3), reasonable (at least one record of VAS 4-6), or bad (at least one record of VAS≥7) during the median IV sufentanil infusion period. Non-steroidal anti-inflammatory drugs (NSAIDs); Otorhinolaryngology (ORL); MW: Mann–Whitney; X^2^: Chi-square; Visual Analogue Scale (VAS); American Society of Anesthesiologists (ASA) physical status. *Statistically significant

Patients with adverse effects related to IV sufentanil infusion	Yes (n=144)		No (n=160)		p-value	Test
Age (median (range), years)	67 (32-91)		64 (22-86)		0.004*	MW
Gender (n %)						
Female	43	(29.9%)	32	(20.0%)	0.062	X^2^
Male	101	(70.1%)	128	(80.0%)		
ASA physical status (n %)						
II	42	(30.9%)	52	(32.5%)	0.824	Fisher
III	98	(64.2%)	104	(65.0%)		
IV	4	(4.9%)	4	(2.5%)		
Previous opioid consumption (n %)						
Yes	24	(16.7%)	14	(8.8%)	0.055	X^2^
No	120	(83.3%)	146	(91.3%)		
Type of surgery (n %)						
Abdominopelvic	59	(41.0%)	64	(40.0%)	0.640	Fisher
Head and neck/ORL	71	(49.3%)	84	(52.5%)		
Thoracic	10	(6.9%)	8	(5.0%)		
Musculoskeletal	3	(2.1%)	1	(0.6%)		
Spine	1	(0.7%)	3	(1.9%)		
IV sufentanil infusion-based protocol (n %)						
With NSAIDs	47	(32.6%)	63	(39.4%)	0.234	X^2^
Without NSAIDs	97	(67.4%)	97	(60.6%)		
Days with IV sufentanil infusion (median (range))	2 (1-13)		1 (1-9)		0.001*	MW
Efficacy of analgesia						
*At rest* (n %)						
Good	117	(95.1%)	152	(98.1%)	0.351	Fisher
Reasonable	5	(4.1%)	3	(1.9%)		
Bad	1	(0.8%)	0			
*At movement* (n %)						
Good	99	(80.5%)	145	(93.5%)	0.243	Fisher
Reasonable	23	(18.7%)	10	(6.5%)		
Bad	1	(0.8%)	0			

Of all adverse effects registered, sedation (n= 104, 42.8%) followed by hypotension (n=32, 13.2%), hypoxemia (n=31, 12.8%), and nausea/vomiting (n=25, 10.3%) were the most common. The majority of adverse effects (n=237, 98.3%) occurred during the first 72 hours of infusion and were mainly managed with opioid dose reductions and did not require specific treatment. Respiratory depression occurred in nine of 144 patients (6.3%), representing, in our sample, an incidence of 2.9%. Only three patients (1%) required advanced treatment: two patients (0.7%) needed naloxone administration and in one patient (0.3%) urgent tracheal intubation and mechanical ventilation were mandatory. No cardiac arrest event was registered.

Further analyzing the group with adverse effects (n=144) related to IV sufentanil infusion, by type of surgery, there is no statistical difference concerning the type of adverse effect, age, ASA physical status classification, or period of infusion. Similar to the whole sample of patients, in this group, patients submitted to musculoskeletal surgery showed the worse efficacy of analgesia (p<0.003) and a higher prevalence of previous opioid consumption (p<0.005).

## Discussion

In this study, we present our institutional experience with IV sufentanil infusions for the management of postoperative acute pain in cancer surgery. Compared to sparse literature with postoperative sufentanil infusions [8,9,12], presently we document the use of conventional infusion pumps with longer patient follow-up. Patient-controlled analgesia (PCA) has well-defined advantages concerning patient pain control [2,13], however, this technology presupposes patients having adequate cognitive function besides the equipment being expensive and not widely available. According to its purpose, IV sufentanil infusions-based protocols were applied mostly in patients submitted to head and neck/ORL and abdominopelvic surgeries.

In terms of the efficacy of analgesia, we found that 90% of the patients had VAS≤3 until the median time with infusion, considering pain at rest and with movement, with the exception of musculoskeletal and spine surgery groups. Our data showed that patients submitted to musculoskeletal surgery were older (p<0.05), presented a higher prevalence of previous opioid consumption (p<0.005) and had higher ASA physical status (p<0.003). However, the small number of patients in these groups could not be representative to make conclusions about the efficacy of IV sufentanil infusions in these types of surgeries.

Regarding adverse effects related to IV sufentanil infusion, we found an incidence of 47.4% of patients who had at least one adverse effect related to IV sufentanil infusion. Mostly did not require specific treatment and occurred during the first three days of infusion. The most common were sedation, transient hypotension, and hypoxia. Adverse effects were more prevalent in older patients and with longer periods of infusion. Older patients are described in the literature as being more sensitive to opioids meanwhile aging-related alterations in renal and hepatic functions [14] predispose to opioid accumulation and may justify this finding.

Because of the risk of excess sedation and respiratory depression, patients who receive systemic opioids for postoperative analgesia should be monitored closely after surgery. Such monitoring should include assessments of alertness and signs or symptoms of hypoventilation or hypoxia with regular observation of respiratory rate and mental status. Although pulse oximetry is frequently used to monitor both oxygenation and ventilation in the postoperative period, it has low sensitivity for hypoventilation, particularly when supplemental oxygen is being administered, and end-tidal CO2 might be more sensitive in promptly identifying respiratory depression [15].

We reported an incidence of 2.9% of respiratory depression associated with sufentanil IV infusion. Most of the cases were mild, did not require specific treatment, and were managed with opioid dose reductions. However, three patients (1%) required advanced treatment: naloxone administration (n=2, 0.7%) and tracheal intubation and mechanical ventilation (n=1, 0.3%). No cardiac arrest event was registered. All the adverse effects were promptly detected and managed which reinforces the importance of proper monitoring and surveillance of patients with IV sufentanil infusions in high-dependency units. Opioid-induced respiratory depression’s incidence is widely variable (0%-46%) according to its definition and evaluation [8,9,12,13,16,17]. Considering the scarce literature focusing on sufentanil-induced respiratory depression, our incidence is higher compared to the ones reported with sufentanil IV PCA (0%) [8,9,12]. Moreover, it is not significantly different from the incidence reported when using IV opioid infusions in general (2%) [13].

Limitations

Due to the study design, this study has some limitations that should be pointed out. First, despite the statistical differences found, due to the small number of patients submitted to musculoskeletal surgery, this could not be representative of the group, limiting the interpretation of our results. Second, we assume a cause-effect relation between IV sufentanil infusion and the adverse effects recorded. However, other external variables, not taken into account such as central nervous system (CNS) depressants administered simultaneously, could contribute. Third, the records found did not permit us to evaluate the total daily dose of sufentanil administered, with which it would have been extremely interesting to confront our adverse effects. Despite these limitations, many of which are inevitable in observational research, we believe that our study has several strengths. We enrolled more than 300 subjects in a period of one year using conventional infusion pumps with longer patient follow-up. Our results may have clinical significance.

## Conclusions

This study provided clinical evidence in favor of IV sufentanil infusions for postoperative multimodal analgesia in cancer surgery. Multimodal analgesic protocols with IV sufentanil infusions provided good postoperative analgesia for head and neck/ORL and abdominopelvic cancer surgeries. Despite the analgesic efficacy, almost half of the patients had at least one adverse effect related to the IV sufentanil infusion, mainly mild and managed with opioid dose reductions, which highlights the importance of continuous and appropriate surveillance in high-dependency units. IV sufentanil infusions can be a safe option for postoperative multimodal analgesia in cancer surgery with appropriate monitoring. Moreover, this study points out the need for higher vigilance in elderly patients and with longer periods of infusion, in order to guarantee both efficacy and safety. In the future, more studies should address the use of IV sufentanil infusions in other types of surgeries.
